# Regional Variations in Coronavirus Disease 2019 Mortality in Japan: An Ecological Study

**DOI:** 10.31662/jmaj.2023-0052

**Published:** 2023-09-27

**Authors:** Akihisa Nakamura, Kazuhiko Kotani, Shuji Hatakeyama, Senichi Obayashi, Ryozo Nagai

**Affiliations:** 1 Division of Community and Family Medicine, Center for Community Medicine, Jichi Medical University, Shimotsuke, Japan; 2 Division of General Internal Medicine, Jichi Medical University Hospital, Shimotsuke, Japan; 3 Division of Infectious Diseases, Jichi Medical University Hospital, Shimotsuke, Japan; 4 Jichi Medical University, Shimotsuke, Japan

**Keywords:** Regional characteristics, COVID-19 mortality, physicians, hospital beds, ecological study, Japan

## Abstract

**Introduction:**

As the characteristics of coronavirus disease 2019 (COVID-19) vary across regions and countries, the relationship between regional characteristics, such as the distribution of physicians and hospital beds, and COVID-19 mortality was assessed in the 47 prefectures of Japan.

**Methods:**

This ecological study was based on the number of patients with COVID-19 by prefecture during the seventh wave of COVID-19 in Japan (June-October 2022). COVID-19 mortality was indexed as the number of COVID-19 deaths divided by the number of new COVID-19 cases. Data on regional factors, such as population size, number of physicians, and hospital beds by prefecture, were obtained from government statistics. Correlations between regional characteristics and COVID-19 mortality index were analyzed by dividing the 47 prefectures into two groups at the median level of population size (more populated group [MPG] ≥ 1.6 million and less populated group [LPG] < 1.6 million).

**Results:**

The COVID-19 mortality index (mean 12.7, minimum-maximum: 4.7-25.7) was correlated negatively with the number of physicians per hospital bed (r = −0.386, *p* = 0.007) and positively with the number of long-term care facilities per 10,000 population (r = 0.397, *p* = 0.006) and aging rate (the proportion of population aged ≥ 65 years) (*r* = 0.471, *p* = 0.001). The two groups varied with respect to the number of physicians (28.7 physicians in the LPG vs. 26.1 physicians in the MPG, *p* = 0.038) and hospital beds (156 beds in the LPG vs. 119 beds in the MPG, *p* < 0.001) per 10,000 population. In the multiple regression analysis, the COVID-19 mortality index was correlated negatively with the number of physicians per hospital bed (*β* = −0.543, *p* = 0.024) and positively with the aging rate (*β* = 0.434, *p* = 0.032) in the LPG, with nonsignificant correlations in the MPG.

**Conclusions:**

The data may suggest a need of improvement in the distribution of physicians and hospital beds in the healthcare system in regions with smaller and older populations to reduce the rate of COVID-19.

## Introduction

Coronavirus disease 2019 (COVID-19) is an infectious disease caused by severe acute respiratory syndrome coronavirus-2 (SARS-CoV-2) ^(1), (2), (3)^. It can cause severe viral pneumonia with respiratory failure that results in death ^(1), (2)^. COVID-19 has been declared as a pandemic ^(4)^, and as of February 28, 2023, approximately 6.8 million deaths have been reported worldwide ^(5)^. In Japan, the number of new COVID-19 cases drastically increased during the seventh wave of COVID-19 from June to October 2022 ^(5), (6)^. The seventh wave was a pandemic of Omicron variant BA.5 ^(7)^. At the peak of this wave, the number of new cases per week in Japan reached 1.49 million ^(6)^, accounting for 21% of the total number of new cases worldwide per week ^(5)^. Under these conditions, 14,805 deaths caused by COVID-19 have been confirmed in Japan ^(6)^. Even before the COVID-19 pandemic, Japan has been facing the challenges of a super-aged society ^(8), (9)^, unrestricted access to medical institutions (a patient can visit any medical institution of their choice without a referral letter, including tertiary hospitals) ^(10), (11)^, and shortage of physicians ^(12)^. The COVID-19 pandemic has promoted the momentum for developing a more efficient healthcare system ^(13), (14)^.

Given the manner of COVID-19 transmission ^(15)^, regional characteristics, such as the distribution of physicians and hospital beds, can also play important roles in COVID-19-related outcomes ^(16), (17)^. A regional healthcare policy is planned at the prefectural level in Japan, which consists of 47 prefectures ^(11), (18)^. Because regional healthcare policies are unique to each prefecture, there are regional differences in the distribution of physicians and hospital beds ^(11), (19)^. These 47 prefectures include certain large regions, such as Tokyo, Osaka, and Aichi, and certain smaller regions with a population of less than one million ^(20)^. This ecological study was conducted to examine the relationship between regional characteristics and COVID-19 mortality.

## Materials and Methods

### Study design and data collection

This ecological study was conducted based on the numbers of COVID-19 new cases and deaths by prefecture during the seventh wave of the pandemic from June 22 to October 18, 2022 ^(21)^. In this study, the COVID-19 mortality index was defined as the number of COVID-19 deaths divided by the number of COVID-19 new cases during the study period multiplied by 10,000. The number of physicians per hospital bed ^(22), (23), (24)^, number of long-term care facilities ^(25), (26), (27)^, and aging rate ^(28), (29)^ were used as regional characteristics in this study. The number of physicians per hospital bed was used as a measure of the distribution of physicians and hospital beds in the healthcare system. Aging rate was defined as the proportion of population aged ≥65 years. The numbers of new COVID-19 cases and deaths by prefecture were obtained from the information provided by the Ministry of Health, Labour, and Welfare ^(6)^. Furthermore, the number of physicians by prefecture was obtained from the 2020 Statistics of Physicians, Dentists, and Pharmacists ^(30)^. The number of hospital beds by prefecture was obtained from the 2021 Survey of Medical Institutions ^(19)^. The number of long-term care facilities by prefecture was obtained from the 2020 Survey of Institutions and Establishments for Long-Term Care ^(31)^ and the 2020 Survey of Social Welfare Institutions ^(32)^. In addition, the population and aging rates by prefecture were obtained from the 2020 Population Census of Japan ^(20)^.

This study was conducted using publicly available government statistics, and personal information was not addressed. Therefore, this study did not require approval from the institutional review board.

### Statistical analyses

The parameters in each prefecture were averaged while expressing the results of this study. Univariate and multiple linear regression analyses were conducted using the number of physicians per hospital bed, number of long-term care facilities per 10,000 persons, and aging rate by prefecture as independent variables and the COVID-19 mortality index as dependent variable. Furthermore, multiple linear regression analyses were conducted using the forced entry method for all three independent variables. Subsequently, stratified analysis was conducted by dividing the 47 prefectures into two groups at the median level of population size (more populated group ≥ 1.6 million and less populated group < 1.6 million). The parameters of each prefecture between the less and more populated groups were compared using the two-sample *t*-test. SPSS version 27 (IBM, Armonk, NY, USA) was used for all statistical analyses, and the significance levels were two-tailed. A *p*-value <0.05 indicated statistical significance.

## Results

**Figure 1.**
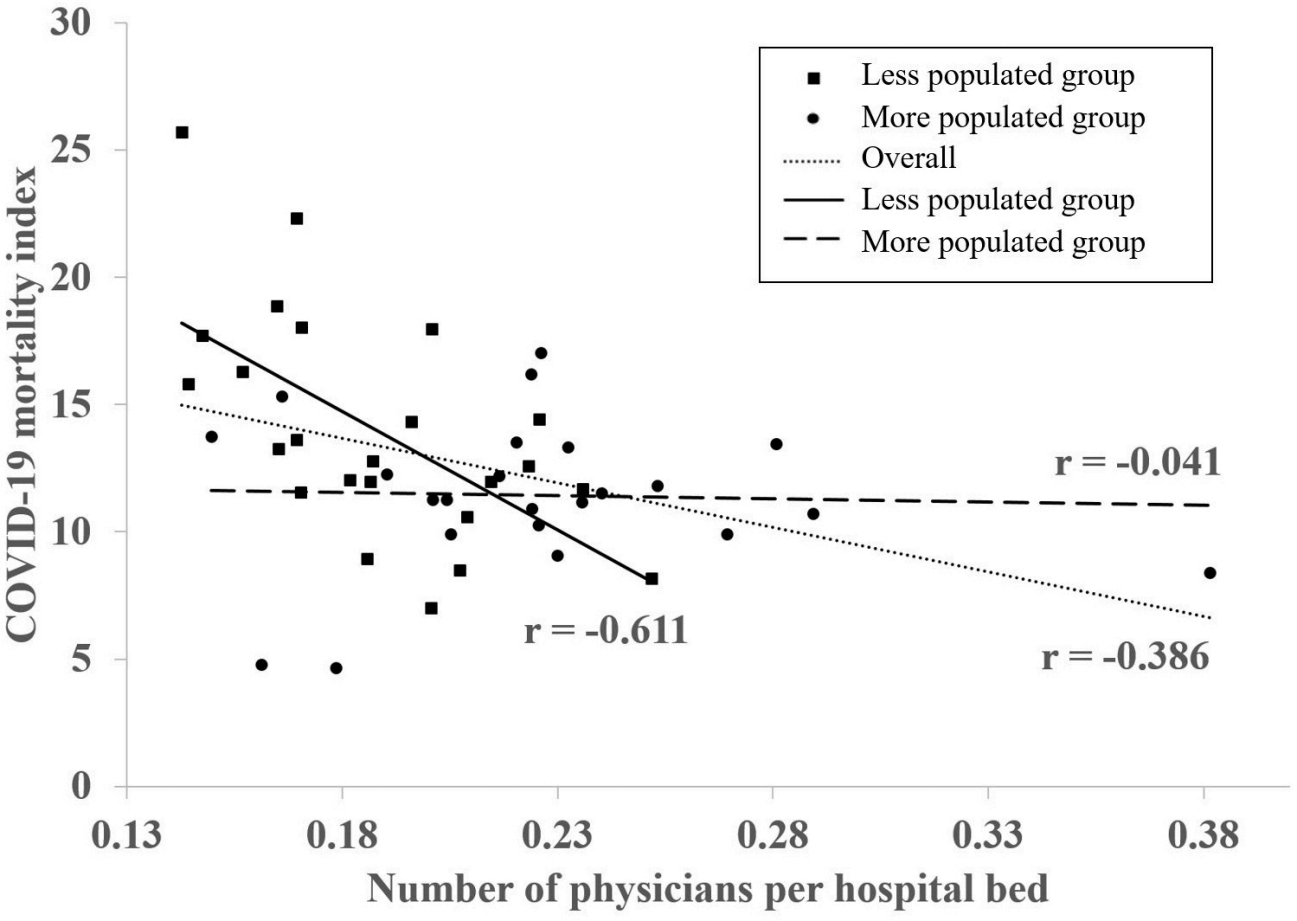
Association between the number of physicians per hospital bed and the COVID-19 mortality index COVID-19: coronavirus disease 2019, *r*: correlation coefficient.

The mean COVID-19 mortality index for the 47 prefectures during the seventh wave of COVID-19 in Japan was 12.7 (minimum-maximum: 4.7-25.7). Up to a 5.5-fold difference in the COVID-19 mortality index was observed among the prefectures. The mean values for each regional characteristic are presented in Table 1. In the univariate analysis, the COVID-19 mortality index was correlated negatively with the number of physicians per hospital bed (*r* = −0.386, *p* = 0.007) (Table 2) (Figure 1) and positively with the number of long-term care facilities per 10,000 persons and the aging rate (*r* = 0.397, *p* = 0.006; *r* = 0.471, *p* = 0.001, respectively). Multiple linear regression analyses revealed a nonsignificant correlation between the regional factors and the COVID-19 mortality index (Table 2).

**Table 1.** COVID-19 Mortality Index and Regional Characteristics (Japan).

	Overall	Less populated group (<1.6 million) (n = 24)	More populated group (≥1.6 million) (n = 23)	*p*-value
COVID-19 mortality index, mean (SD)	12.7 (4.0)	14.0 (4.9)	11.4 (3.0)	0.026^※^
Number of physicians per hospital bed, mean (SD)	0.21 (0.04)	0.19 (0.03)	0.23 (0.05)	0.002^※^
Number of physicians per 10,000 population, mean (SD)	27.4 (4.3)	28.7 (3.6)	26.1 (4.7)	0.038^※^
Number of hospital beds per 10,000 population, mean (SD)	137 (35)	156 (30)	119 (28)	<0.001^※^
Number of LTCF per 10,000 population, mean (SD)	4.29 (1.4)	5.12 (1.3)	3.42 (1.0)	<0.001^※^
Aging rate (≥65 y) (%), mean (SD)	30.7 (3.1)	32.3 (3.0)	29.1 (2.4)	<0.001^※^

COVID-19: coronavirus disease 2019, SD: standard deviation, LTCF: long-term care facilities ^※^*p* < 0.05

**Table 2.** Association between Regional Characteristics and COVID-19 Mortality Index in All the 47 Prefectures.

Variables	Univariable		Multivariable^※※^	
	Correlation coefficient	*p*-value	Beta	*p*-value
Number of physicians per hospital bed	−0.386	0.007^※^	−0.053	0.805
Number of LTCF per 10,000 population	0.397	0.006^※^	0.099	0.643
Aging rate (≥65 y) (%)	0.471	0.001^※^	0.362	0.094

COVID-19: coronavirus disease 2019, LTCF: long-term care facilities ^※^*p* < 0.05 ^※※^Multiple linear regression analyses were conducted using the forced entry method for all three independent variables.

Thereafter, the 47 prefectures were divided into less populated and more populated groups at the median level of population size, and the COVID-19 mortality index and regional characteristics were compared between these groups (Table 1). The mean COVID-19 mortality indices in the less and more populated groups were 14.0 (minimum-maximum: 7.0-25.7) and 11.4 (minimum-maximum: 4.7-17.0), respectively. The more populated group had significantly lower mean COVID-19 mortality index than the less populated group (*p* = 0.026). Furthermore, the mean number of physicians per hospital bed was significantly higher in the more populated than the less populated group (*p* = 0.002). The mean number of physicians per 10,000 population in the more populated group (26.1 physicians) was significantly lower than that in the less populated group (28.7 physicians) (*p* = 0.038). In addition, the mean number of beds per 10,000 persons was significantly lower in the more populated group (119 hospital beds) than the less populated group (156 hospital beds) (*p* < 0.001). The mean number of long-term care facilities per 10,000 persons and the mean aging rate were significantly lower in the more populated group than the less populated group (*p* < 0.001 and *p* < 0.001, respectively). In the stratified multiple linear regression analyses (forced entry method) for the less populated group, the COVID-19 mortality index was correlated negatively with the number of physicians per hospital bed (*β* = −0.543, *p* = 0.024) and positively with the aging rate (*β* = 0.434, *p* = 0.032) (Table 3). In the multiple linear regression analyses for the more populated group, a nonsignificant correlation was observed between the regional factors and COVID-19 mortality index.

**Table 3.** Association between Regional Characteristics and COVID-19 Mortality Index According to Population Size.

Variables	Univariable		Multivariable^※※^	
	Correlation coefficient	*p*-value	Beta	*p*-value
Less populated group (<1.6 million) (n = 24)
Number of physicians per hospital bed	−0.611	0.002^※^	−0.543	0.024^※^
Number of LTCF per 10,000 population	0.352	0.091	−0.231	0.304
Aging rate (≥65 y) (%)	0.601	0.002^※^	0.434	0.032^※^
More populated prefectures (≥1.6 million) (n = 23)
Number of physicians per hospital bed	−0.041	0.852	−0.139	0.628
Number of LTCF per 10,000 population	0.098	0.656	0.293	0.409
Aging rate (≥65 y) (%)	−0.053	0.811	−0.432	0.334

COVID-19: coronavirus disease 2019, LTCF: long-term care facilities ^※^*p* < 0.05 ^※※^Multiple linear regression analyses were conducted using the forced entry method for all three independent variables.

## Discussion

In this study, a 5.5-fold difference was observed in the COVID-19 mortality index by region. Such differences have also been observed in several regions ^(22), (23), (33), (34)^. For example, a previous study in 30 European countries reported up to 185-fold regional differences in the COVID-19-related deaths among these nations ^(34)^. Another previous study in Italy reported differences of up to 9.9-fold in COVID-19 mortality among regions ^(22)^; similar results were observed in Japan. That Italian study reported a negative correlation between COVID-19-related deaths and the number of physicians; furthermore, a positive correlation was observed between COVID-19-related deaths and the number of hospital beds ^(22)^. Several previous studies have shown a negative correlation between COVID-19 mortality and the number of physicians ^(22), (23), (24)^. However, there was an inconsistent trend in the relationship between the number of hospital beds and COVID-19 mortality ^(22), (23), (24), (35)^.

In this study, the number of physicians per hospital bed was not significantly correlated with the COVID-19 mortality index in the more populated group and was negatively correlated with the COVID-19 mortality index in the less populated group. This finding indicates that there were fewer COVID-19-related deaths in cases where the number of physicians per hospital bed was higher in regions with a smaller population. Different results were obtained for the less and more populated groups, which could be explained by some speculations.

First, the differences in the number of physicians per hospital bed between the two groups should be considered. The number of physicians per hospital bed was lower in the less populated group than the more populated group. Of all the members of the Organization for Economic Cooperation and Development (OECD), Japan has the highest number of hospital beds per 1,000 population (12.8 hospital beds/1000 population) (OECD average: 4.4 hospital beds/1,000 population); also, the number of physicians per 1,000 population (2.5 physicians/1,000 population) in Japan is well below the OECD average (3.6 physicians/1,000 population) ^(36)^. Therefore, it has been dealing with a large number of hospital beds with a relatively few medical staff. In the less populated group, this situation could have been more pronounced, increasing the burden per physician and probably resulting in an increase in the number of COVID-19-related deaths. Second, the differences in the number of hospital beds between the two groups should also be considered. The less populated group had more hospital beds than the more populated group. Hospital inpatients are often older adults or patients with underlying medical conditions; they are known to be vulnerable to nosocomial infection with COVID-19 and have a poor prognosis ^(37), (38), (39)^. In Japan, nosocomial outbreaks were confirmed in many regions during the seventh wave of COVID-19 ^(40), (41)^. Thus, the less populated group had more hospital beds and was at an increased risk of exposure to nosocomial infections, which might have resulted in more COVID-19-related deaths.

In this study, COVID-19 mortality and aging rate had a nonsignificant correlation in the more populated group but a positive correlation in the less populated group. Older adults have been reported to be at an increased risk for COVID-19-related death ^(28), (29)^, and the aging rate was higher in the less populated than the more populated group. Thus, COVID-19-related deaths may have been more common in Japan in regions with a large elderly population.

Japan has the highest aging rate in the world, which is expected to further increase in the future ^(8), (11)^. This rising aging rate has promoted a momentum for the development of an efficient healthcare system; this healthcare system reform is called Regional Medical Care Visions in Japan ^(11), (42)^. The distribution of physicians and hospital beds could be possibly organized while strengthening the cooperation among medical institutions and between medical and nursing care as well as enhancing home medical care under this reform initiative ^(11), (42)^. In this study, the number of physicians per hospital bed was negatively correlated with COVID-19 mortality in the less populated group. The findings of this study suggest a potential need for organizing the distribution of physicians and hospital beds in the healthcare system in regions with smaller populations, even regarding COVID-19 measures.

The strength of this study was the high reliability of the results as government statistical data were used. Analysis was conducted for units of the 47 prefectures, which allowed the use of government statistics. Nevertheless, this study has several limitations. First, this is an observational study with prefectures used as the unit of observation and therefore cannot demonstrate causal relationships. Second, the risk of death among Omicron-positive individuals is known to be age-dependent ^(43)^; however, in this study, age adjustment could not be performed for the COVID-19 mortality index because the ages of patients who died due to COVID-19 were not publicly available in approximately one-third of the prefectures. Third, factors such as climate and the number of international travelers to Japan are known to be related to COVID-19 mortality ^(33), (44)^; however, detailed regional data on these factors were not considered in this study. Fourth, this study analyzed the seventh wave, when Omicron variant BA.5 was predominant ^(7)^; however, whether similar results will be obtained in future pandemics may depend on the emergence of mutant strains and regional conditions.

In conclusion, COVID-19 mortality was negatively correlated with the number of physicians per hospital bed and positively with the aging rate in the less populated group. These findings suggest a potential need for organizing the distribution of physicians and hospital beds in the healthcare system in regions with a smaller and older population, even regarding COVID-19 measures.

## Article Information

### Conflicts of Interest

None

### Sources of Funding

This work was supported by [KAKENHI] grant number [JP21K17230] from the Japan Society for the Promotion of Science (JSPS).

### Acknowledgement

The authors are grateful to the Japan Society for the Promotion of Science (JSPS) for providing a grant to conduct this study.

### Author Contributions

RN and SO conceived the study. AN and KK contributed to the study design. AN collected and analyzed the data and wrote the original graft. KK, SH, SO, and RN critically revised the manuscript. RN supervised the study. All the authors approved the final manuscript.

### Approval by Institutional Review Board (IRB)

This study was conducted using publicly available government statistics, and personal information of the participants was not addressed. Therefore, this study did not require approval from the institutional review board of the respective institution.
